# Case Report: Ludwig's angina - 'The Dangerous Space'

**DOI:** 10.12688/f1000research.127242.2

**Published:** 2024-06-27

**Authors:** Satish Vasanth, Satheesh Chandran, Deepak Abraham Pandyan, Padmashini Gnanam, Sinouvassane Djearamane, Ling Shing Wong, Siddharthan Selvaraj

**Affiliations:** 1 Department of Oral And Maxillofacial Surgery, Faculty of Dentistry, AIMST University, Bedong, Kedah, 08100, Malaysia; 2 Department of Oral and Maxillofacial Surgery, Madha Dental College and Hospital, Chennai, 600069, India; 3 Department of Prosthodontics, Faculty of Dentistry, AIMST University, Bedong, Kedah, 08100, Malaysia; 4 Department of Biomedical Science, Faculty of Science, Universiti Tunku Abdul Rahman, Kampar, Perak, 31900, Malaysia; 5 Faculty of Health and Life Sciences, INTI International University, Nilai, 71800, Malaysia; 6 Department of Community Oral Health and Clinical Prevention, Faculty of Dentistry, University of Malaya, 50603, Kuala Lumpar, Malaysia

**Keywords:** Ludwig's angina, odontogenic space infection, surgical decompression, incision, drainage, case report

## Abstract

**Background:** Ludwig's angina is a potentially life-threatening disease characterized by diffuse bilateral cellulitis with an odontogenic origin. This unique infection is now rare owing to the antibiotic era.

**Case:** This patient presented to the emergency room with trismus, jaw and neck swelling, mild respiratory distress with tachypnea, hyperthermia, and panic. Clinical examination and radiographic evaluation confirmed the diagnosis of Ludwig's angina. As it is a quickly spreading infection, the patient was taken up for immediate surgical decompression leading to pus drainage, removal of the offending tooth, bacterial culture and sensitivity, and administration of empirical antibiotics. As we had operated promptly, there was no need for emergency airway intervention, and the patient had immediate relief from airway distress.

**Conclusions:** Early accurate diagnosis with conservative surgical decompression, thereby negating the need for airway intervention, was vital to avoiding mortality which is always possible in such an expeditious infection.

## Introduction

Ludwig's angina, diffuse cellulitis on the floor of the mouth, was first described by a German physician, Wilhelm Frederick von Ludwig, in 1836. ^1^ The old terminology describes it as “ *angina maligna*” in Latin ( *angere*-to strangle, *Morbus strangularis*). ^2^ Ironically, Ludwig passed away at 75 from the same illness he had earlier described. ^3^ The most prevalent etiology seems odontogenic in origin. ^4^ Systemic illnesses such as diabetes mellitus, malnutrition, alcoholism, and AIDS may be risk factors. ^5^ The incidence has recently dropped from 60% to 10% due to antibiotics and better oral hygiene practices. ^2^ Although there are no current guidelines for managing acute Ludwig's angina, the mainstay of the treatment includes airway maintenance and following the principles of infection control which we have discussed in this case report. The objective of this case report was to raise awareness and facilitate the detection of similar occurrences, given their rarity.

## Case report

**Figure 1.**
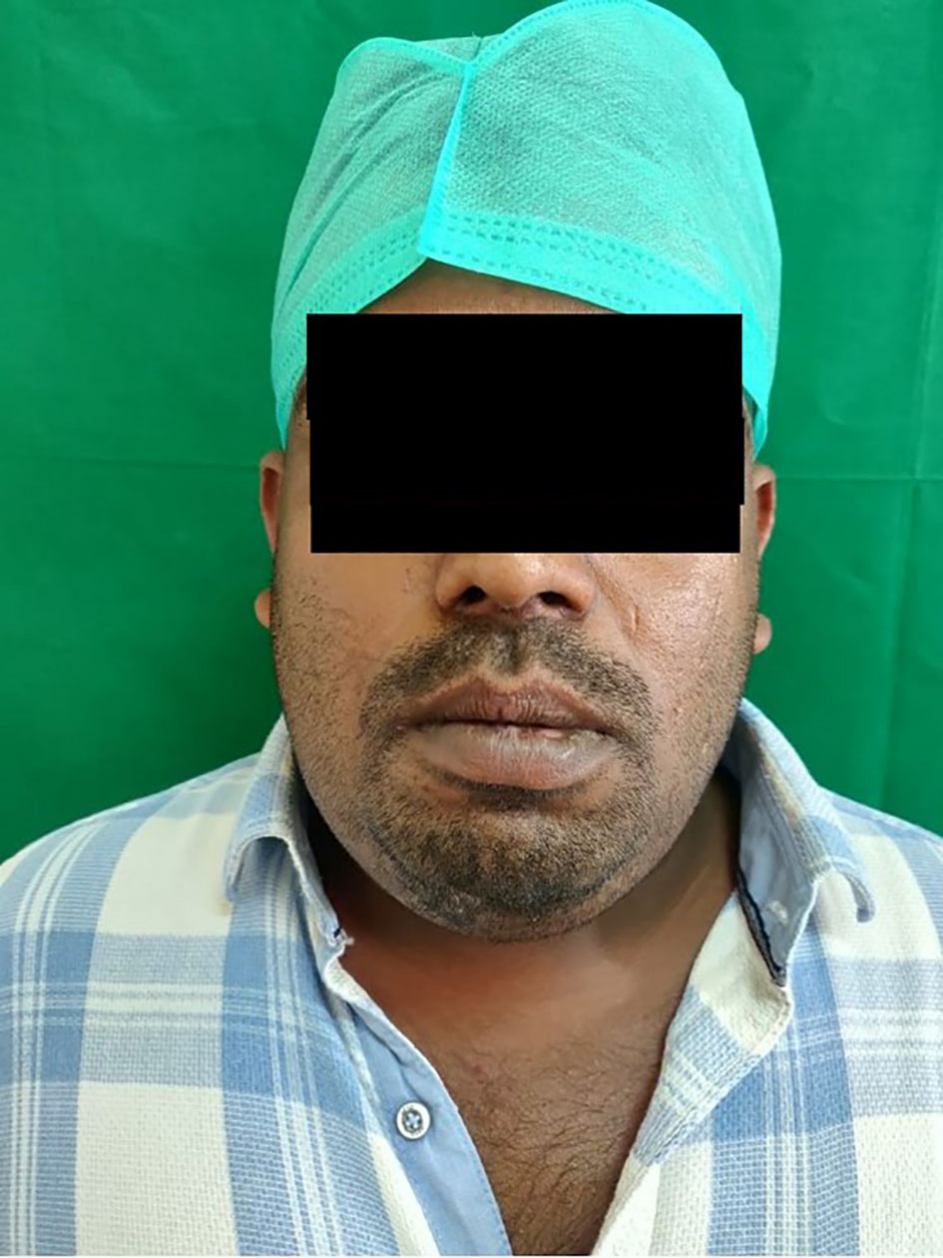
Patient with prominent swelling in the submandibular region.

A 40-year-old Indian male patient working as an electrician reported to the department of Oral and Maxillofacial Surgery with a chief complaint of inability to open the mouth, along with pain and swelling in the lower jaw and neck region for the past three days [ Figure 1].

**Figure 2.**
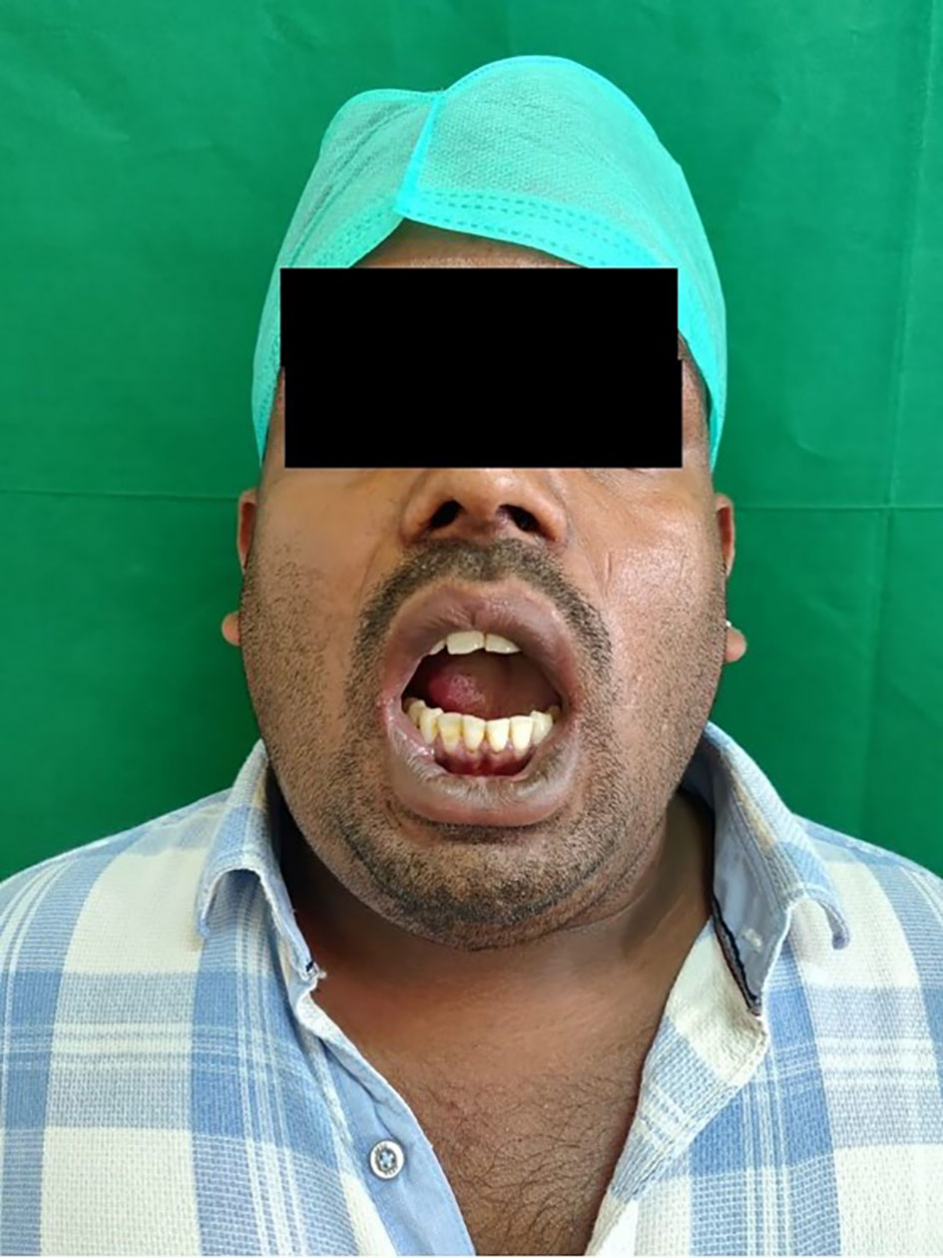
Elevation and posteriorly displaced tongue.

**Figure 3.**
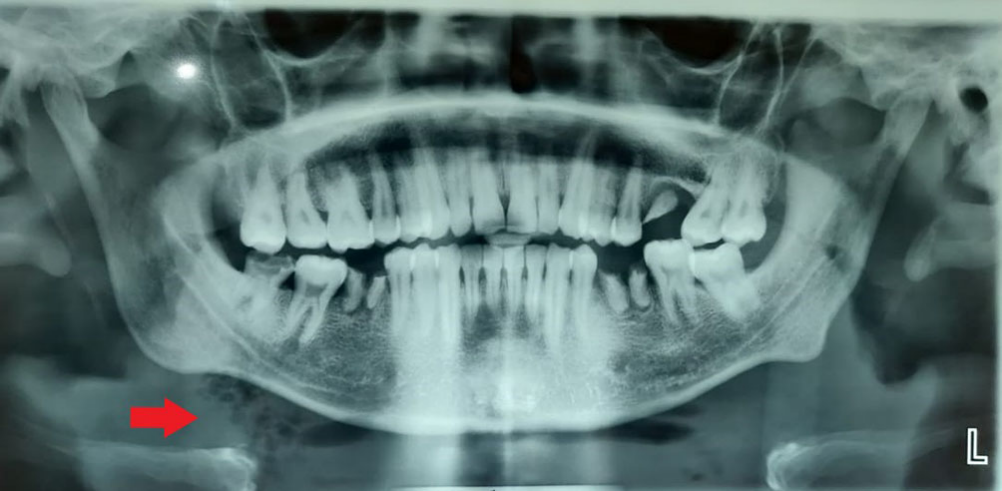
Presence of air/pus locules in the right submandibular region.

No relevant medical history was noted. The patient reported hyperthermia (100° F), pulse rate of 99 beats per minute, respiratory rate of 25 breaths per minute, and oxygen saturation fluctuating between 88 to 90 SPO _2_. On clinical examination, he reported a restricted mouth opening of only 19 mm (about 0.75 in), a tongue deviation [ Figure 2], and evident dysphagia and dyspnea. Extra orally, the swelling was indurated, non-fluctuant, with bilateral involvement of the submandibular and sublingual spaces. His dentate status revealed root stumps in relation to 36 and 46 regions, which were tender on percussion. The obliteration of the mucogingival junction was also noted. Radiographic examination with orthopantomogram (OPG) revealed periapical radiolucency concerning 36, 37 and 38 with locules in the submandibular region (more on the right side than the left side), indicating air or pus [ Figure 3].

**Figure 4.**
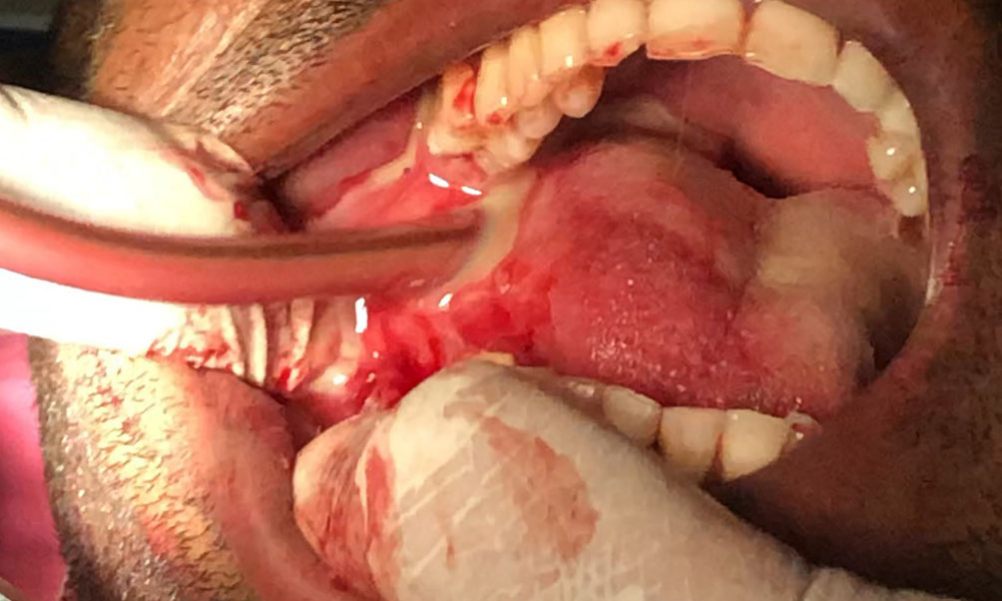
Lingual vestibular Incision given, and pus evacuated intraorally.

**Figure 5.**
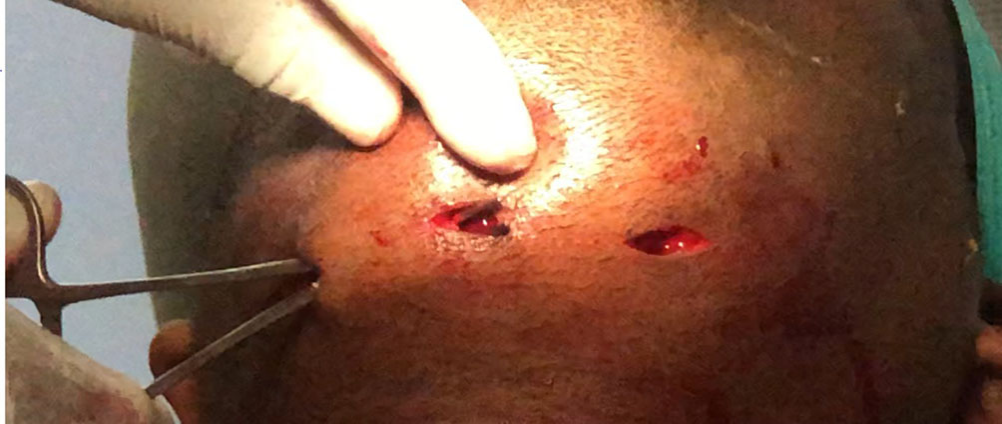
Extra oral incisions.

It was diagnosed as Ludwig's angina, and Hilton's incision and drainage were administered promptly. Separate stab incisions were placed with the No. 11 BP blade on the bilateral submandibular and submental regions. The bilateral submandibular spaces were connected to the midline through blunt dissection. The root stumps were extracted with the evacuation of inflammatory exudate. An intra-oral incision was made along the lingual sulcus in relation to 36 and 37, along with two extraoral incisions in the submandibular region combined with superficial dissection; the pus was evacuated [ Figures 4 and 5] along with copious povidone-iodine and saline irrigation. Radiographic examination with orthopantomogram (OPG) revealed periapical radiolucency concerning 36, 37 and 38 with locules in the submandibular region (more on the right side than the left side), indicating air or pus. The pus was collected with a sterile swab and sent for culture and antibiotic sensitivity test. The culture sensitivity report revealed the sensitivity to prescribed empirical antibiotics; therefore, the same medicines were continued for a week.

**Figure 6.**
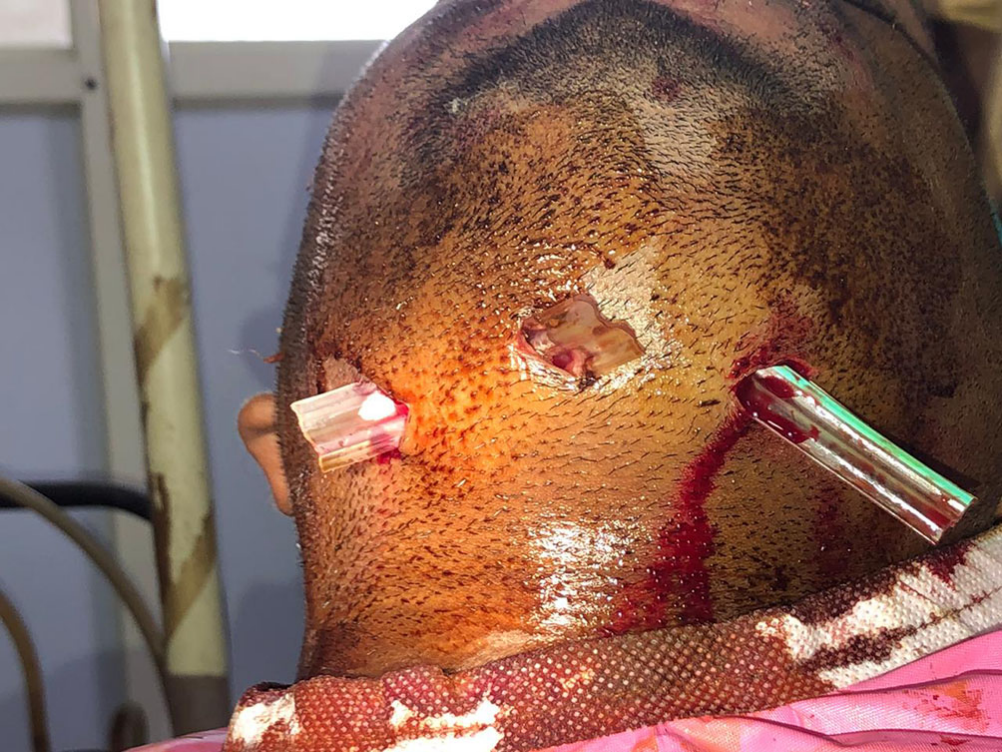
Placement of rubber drains.

Extra orally corrugated rubber drain tubes were positioned and secured to the skin with silk sutures [ Figure 6]. The patient subjectively experienced a reduction in dyspnea. The pus was collected with a sterile swab and sent for culture and antibiotic sensitivity test. The patient was prescribed empirical antibiotics of Tablet amoxicillin and potassium clavulanate 625 mg twice daily, Tablet metronidazole 400 mg thrice daily for five days orally, and the drain tubes were disconnected after five days. Furthermore, maintaining patent airway is the most important consideration, removing the source of infection, antibiotics and Incision and drainage would be course of action.

**Figure 7.**
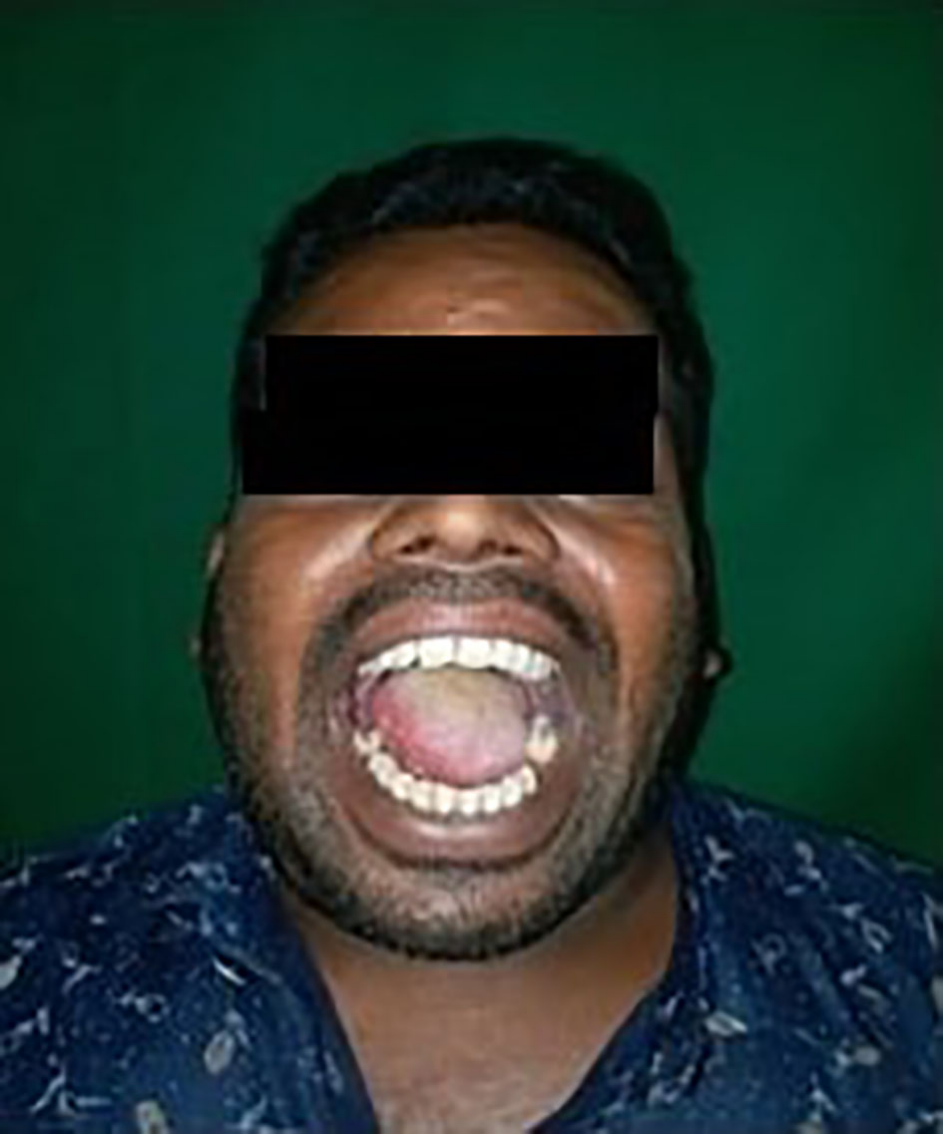
Post operative results after 1 week.

The culture sensitivity report revealed the sensitivity to prescribed empirical antibiotics; therefore, the same medicines were continued for a week. After one week, the patient's re-evaluation revealed adequate wound healing and an increased mouth opening of 36 mm (about 1.42 in). The infection had nearly resolved with the disappearance of the symptoms. Post operative results showed adequate mouth opening and the infection was resolved [ Figure 7].

## Discussion

Ludwig's angina is a plausible life-threatening connective tissue infection of the floor of the mouth and neck, characterized by bilateral brawny supra hyoid swelling over bilateral sublingual and submandibular spaces. It can either lead to airway obstruction or, at a later stage, cause dissemination of the infection, which then spreads through several other adjacent spatial planes. ^6^ The most cultured organisms include staphylococcus, streptococcus, and Bacteroides species of either aerobic or anaerobic origin. ^1^ Ludwig's angina is often correlated with the region between the second and third lower molars. Since the apices of these teeth are positioned directly below the mylohyoid ridges, the submandibular space is proximal. If these teeth are infected, the infection will progress and perforate the lingual plate. The infection may rapidly spread into the submandibular, sublingual, and submental tissue spaces. ^7^ According to the literature, four significant signs indicate the diagnosis of Ludwig's angina: (a) bilateral involvement of more than a single deep-tissue space (b) gangrene with serosanguinous, putrid infiltration but little or no frank pus (c) involvement of connective tissue, fasciae, and muscles but not the glandular structures (d) spread through fascial space continuity rather than by the lymphatic system. ^8^ If not diagnosed early, complications include thrombophlebitis of the internal jugular vein, mediastinitis, empyema, pericardial effusion, osteomyelitis, aspiration pneumonia, and pleural effusion. ^1^

Typically, the infection begins at the floor of the mouth and progresses to the submandibular space rapidly, leading to elevation and posterior displacement of the tongue. ^2^ The physical examination usually reveals aggressive gangrenous cellulitis, including fever, tachycardia, brawny induration and swelling of a tender submandibular space, and an elevated, protruding tongue. Trismus irritates the muscles of mastication. ^7^ The enlargement of the anterior soft tissues of the neck above the hyoid bone sometimes leads to the characteristic “bull's neck” appearance of affected patients. ^8^ Some signs of impending airway distress are hoarseness of voice, stridor, respiratory distress, decreased air movement, cyanosis, and a “sniffing” position (upright posture with the neck being put forward with the chin elevated). ^8^ Patients may lean forward in the tripod posture as respiratory distress intensifies to enhance airway diameter and obtain relief. ^1^

The risk factors such as age over 65 years old, immunocompromised conditions, diabetes mellitus, and alcoholism are said to increase the mortality and complications in Ludwig's angina. In addition, extended hospital stays, diabetes, and hemodialysis increases the risk of methicillin-resistant Staphylococcus aureus (MRSA) infection. ^1^ The parameters used to analyze the risk factors leading to life-threatening complications are age, sex, diabetes, type and side of submandibular involvement, anterior visceral space involvement, type of infection, and symptoms like trismus and fever. According to one study, diabetes mellitus is commonly associated with Ludwig's angina. Logistic regression analysis confirmed the other co-morbidities and bilateral submandibular swelling as predictors for mortality in Ludwig's angina. ^9^ Radiographs of the neck and chest often reveal the degree of soft tissue edema and gas accumulation in the tissues, especially in cases of anaerobic infection. The presence of air in the neck or mediastinum is diagnostic of the intrathoracic extension of the infectious process. ^10^ According to some authors, ultrasonography may be beneficial in diagnosing early cases of abscess and cellulitis. ^5^ In addition, computed tomography (CT) and magnetic resonance imaging (MRI) may be proposed to detect airway edema and identify mediastinal fluid collections. CT is accurate in evaluating deep neck and mediastinal collections of pus. By contrast, MRI generates higher resolution images, but imaging time is longer, so CT is the recommended investigation of choice. ^1^

Although there are no current guidelines for managing acute Ludwig's angina, the mainstay of the treatment includes airway maintenance, where the patency of the airway must be evaluated. The administration of broad-spectrum antibiotics follows evaluation, incision, and drainage removal of the infectious foci, *i.e.*, mandibular molar, pus culture, and sensitivity test. Literature suggests that the inappropriate use of drugs like antibiotics, steroids & nonsteroidal anti-inflammatory drugs may affect the clinical signs and symptoms of infection and slow the progression of the disease, thereby potentially delaying the precise diagnosis of the condition. ^2^ However, these medications are still provided to reduce the risk of airway derailment. ^4^ Despite the use of steroids, most patients in the research (71%) were treated with surgical decompression and drain insertion, demonstrating that steroids are not the primary treatment mechanism. However, it is still debatable whether the usage of steroids necessitates surgical intervention. ^4^ Corticosteroids are reported to decrease facial edema and airway edema, as well as facilitate antibiotic penetration. The steroid of choice is dexamethasone (IV 10 mg). Furthermore, nebulized epinephrine (1 mL of 1:1,000 diluted to 5 mL in 0.9% normal saline) reduces airway blockage: However, evidence is scarce. Despite contradictory findings, early surgical intervention improves airway conditions. ^1^ Penicillin is the antibiotic of choice for empirical antibiotic coverage, which targets gram-positive cocci predominantly. Considering the increasing prevalence of penicillin resistant Bacteroides strains, metronidazole is recommended for the anaerobic cover. ^4^ Clindamycin alone is not advocated, as resistance rates for streptococcal species and MRSA exceed 30%. ^1^

Surgery is usually accomplished by decompressing the submental, sublingual, and submandibular spaces by external incision and drainage. Significant airway compromise, characterized by stridor and the use of accessory muscles for breathing, necessitates a definitive airway, which can be attained *via* endotracheal intubation or tracheostomy, depending on the clinical scenario: Mask breathing will be complicated owing to neck swelling; thus, it is essential to pre oxygenate these patients using any suitable methodology. Blind oral or nasotracheal intubation can culminate in airway damage, increasing edema, and severe laryngospasm; hence, this technique is not advised. Additionally, supraglottic airway devices should be discouraged since they cannot be positioned correctly when the edema progresses. Ideally, patients should be treated with nasotracheal intubation while seated utilizing a flexible intubating endoscope and an awake intubation approach, anticipating a surgical airway ( *i.e.*, cricothyrotomy) if necessary. ^1^ However, emergency cricothyroidotomy or tracheostomy is preferred in patients in the late stages. ^2^ In a retrospective study comparing the conservative (antibiotics alone) and surgical decompression with antibiotics, the conclusion was that the latter is superior as there is always a possibility of antibiotics failure. ^6^ A review of 29 cases over nine years by Greenberg *et al.*, showed that 21 cases (72%) were managed conservatively, one case required emergency intubation, six cases (24%) were intubated using fiberoptic Naso endoscopy, and one case (3%) required tracheostomy under local anesthesia. ^10^ In the present case report, the patient was treated conservatively, with the extraction of the mandibular molar, incision and drainage of the involved spaces, and administration of broad-spectrum antibiotics without airway intervention. One week after surgery, adequate mouth opening was achieved with the support of mouth opening exercises.

In the present case, air pockets in the neck alerted us to the likelihood of a deadly infection progressing fast ^9^^,^^11^ [ Figure 3]. We have attempted to eliminate the observer's subjective bias by employing many well-trained observers to document the case independently. In the research about Ludwig's angina, there are voids concerning risk factors such as smoking, drinking, and inadequate antibiotic coverage. Additionally, there are no data on this illness in COVID-19 patients, particularly in this pandemic era. According to our research, there needs to be a precise treatment plan and agreement for Ludwig's angina that could serve as a future reference. The literature on steroids' effects on this illness is still ambiguous; therefore, additional research is required.

## Conclusions

The clinician should be familiar with the presentation of Ludwig's angina since prompt diagnosis and investigation, administration of antibiotic therapy, and possible surgical management is required to prevent the associated morbidity and mortality.

### Patient perspective

I am willing to share my experience if it helps others. It only started as a small swelling in my jaw but later became big and made me uncomfortable as breathing became difficult. I should have come before. But thank God I am better now.

### Consent

Written informed consent for publication of their clinical details and clinical images was obtained from the patient.

## Data Availability

All data underlying the results are available as part of the article and no additional source data are required.

## References

[1] Bridwell R Gottlieb M Koyfman A : Diagnosis and management of Ludwig's angina: An evidence-based review. *Am. J. Emerg. Med.* 2021;41:1–5. 10.1016/j.ajem.2020.12.030 33383265

[2] Dowdy RAE Emam HA Cornelius BW : Ludwig's Angina: Anesthetic Management. *Anesth. Prog.* 2019 Summer;66(2):103–110. 10.2344/anpr-66-01-13 31184944 PMC6560692

[3] Wasson J Hopkins C Bowdler D : Did Ludwig's angina kill Ludwig? *J. Laryngol. Otol.* 2006;120(5):363–365. 10.1017/S0022215106000806 16696873

[4] Tami A Othman S Sudhakar A : Ludwig's angina and steroid use: A narrative review. *Am. J. Otolaryngol.* 2020;41(3):102411. 10.1016/j.amjoto.2020.102411 32035654

[5] Vallée M Gaborit B Meyer J : Ludwig's angina: A diagnostic and surgical priority. *Int. J. Infect. Dis.* 2020;93:160–162. 10.1016/j.ijid.2020.01.028 31981767

[6] Edetanlen BE Saheeb BD : Comparison of Outcomes in Conservative versus Surgical Treatments for Ludwig's Angina. *Med. Princ. Pract.* 2018;27(4):362–366. 10.1159/000490740 29886486 PMC6170910

[7] Barakate MS Jensen MJ Hemli JM : Ludwig's angina: report of a case and review of management issues. *Ann. Otol. Rhinol. Laryngol.* 2001;110(5 Pt 1):453–456. 10.1177/000348940111000511 11372930

[8] David M Lemonick MD : Ludwig's Angina: Diagnosis and Treatment. *Hosp. Physician.* 2002;38:31–37.

[9] Snow N Lucas AE Grau M : Purulent mediastinal abscess secondary to Ludwig's angina. *Arch. Otolaryngol.* 1983;109(1):53–55. 10.1001/archotol.1983.00800150057011 6848108

[10] Boscolo-Rizzo P Da Mosto MC : Submandibular space infection: a potentially lethal infection. *Int. J. Infect. Dis.* 2009;13(3):327–333. 10.1016/j.ijid.2008.07.007 18952475

[11] Vasanth S Gnanam P Selvaraj S : Ludwig’s Angina- a Forgotten Crisis in the Field of Dentistry? *J. Dental Sci.* 2022;7(3):000344.

