# Supplementary material for: Case Report: Ludwig's angina - 'The Dangerous Space'
Source: F1000Res. 2024 Jun 27;11:1511. Originally published 2022 Dec 13. [Version 2] doi: 10.12688/f1000research.127242.2 (PMC11408913; doi:10.12688/f1000research.127242.2)
Supplement: Peer review history

# Reviewer report: Reviewer response for version 2

**DOI:** 10.5256/f1000research.166856.r321293

**Published:** 2024-09-17

**Authors:** Marcelo Silva Monnazzi

**Affiliations:** 1 São Paulo State University (Unesp) School of Dentistry (Araraquara), Sao Paulo, Brazil

The cases history and progression could be a little more detailed. 

 But in general I think the article is good. And it is worth indexing.

 I just think that it is necessary to remove the citations that the Ludwig angina is a rare infection, because it is not so rare. At least the authors could state that it is rare in their area (if it was the case), because here in Brazil, it is not rare at all.

Are enough details provided of any physical examination and diagnostic tests, treatment given and outcomes?

Yes

Is the case presented with sufficient detail to be useful for other practitioners?

Yes

Is sufficient discussion included of the importance of the findings and their relevance to future understanding of disease processes, diagnosis or treatment?

Yes

Is the background of the case’s history and progression described in sufficient detail?

Partly

Reviewer Expertise:

Oral and maxillofacial surgeon for 24 years. Professor of oral and maxillofacial at the São Paulo state university for at least 17 years. DR. Msc. Phd.

I confirm that I have read this submission and believe that I have an appropriate level of expertise to confirm that it is of an acceptable scientific standard.

# Reviewer report: Reviewer response for version 1

**DOI:** 10.5256/f1000research.139725.r272616

**Published:** 2024-05-10

**Authors:** Paramjot Kaur

**Affiliations:** 1 Baba Jaswant Singh Dental College, Ludhiana, Punjab, India

The authors have failed to reference any established protocols for the management of Ludwig's angina. What course of action should be pursued if the administration of steroids and epinephrine are contraindicated for a case of Ludwig's angina? The authors have neglected to include citations of recent publications. Is Ludwig's angina the most perilous type of facial and submandibular space infection? Justify the designation of 'dangerous space' in this context. The primary cause for the development of this condition in a 40-year-old male remains unspecified. Which specific diagnostic procedures were employed in the examination of this particular case? No details have been provided regarding the utilization of laboratory tests and radiographic imaging in the assessment. What are the respective roles of the first and second authors in the execution of this research? Was consent obtained from the patient prior to the capture of clinical photographs? The deficiency of comprehensive details in both the case presentation and subsequent deliberations is evident.

Are enough details provided of any physical examination and diagnostic tests, treatment given and outcomes?

No

Is the case presented with sufficient detail to be useful for other practitioners?

Yes

Is sufficient discussion included of the importance of the findings and their relevance to future understanding of disease processes, diagnosis or treatment?

Partly

Is the background of the case’s history and progression described in sufficient detail?

Yes

Reviewer Expertise:

oral and maxillofacial surgery

I confirm that I have read this submission and believe that I have an appropriate level of expertise to confirm that it is of an acceptable scientific standard, however I have significant reservations, as outlined above.

# Response

**Published:** 2024-05-20

**Authors:** Siddharthan Selvaraj

**Affiliations:** Faculty of Dentistry, University of Malaya, Kuala Lumpur, Federal Territory of Kuala Lumpur, Malaysia

Dear Reviewers,

 We would like to thank the reviewers for taking their valuable time to provide their insightful queries, we have provided our explanation for the same. Once again thank you for your feedback. 

 1.The authors have failed to reference any established protocols for the management of Ludwig's angina.

**Answer:  Although there are no current guidelines for managing acute Ludwig's angina, the mainstay of the treatment includes airway maintenance and following the principles of infection control which we have already discussed.**

 2. What course of action should be pursued if the administration of steroids and epinephrine are contraindicated for a case of Ludwig's angina?

**Answer: Maintaining patent airway is the most important consideration. Removing the source of infection, antibiotics and Incision and drainage would be course of action .**

 3.The authors have neglected to include citations of recent publications. Is Ludwig's angina the most perilous type of facial and submandibular space infection?

**Answer: yes, it is highly perilous but in this antibiotic era it has become a rare infection which makes recent publications little difficult.**

 5.Justify the designation of 'dangerous space' in this context.

**Answer:** **It can either lead to airway obstruction or, at a later stage, cause dissemination of the infection, which then spreads through several other adjacent spatial planes.**

 5.The primary cause for the development of this condition in a 40-year-old male remains unspecified.

**Answer: yes the patient wasn’t immuno compromised or medically compromised at the time of diagnosis and it still happened**

 6.Which specific diagnostic procedures were employed in the examination of this particular case?

**Answer: There is no specific diagnostic test which gives the diagnosis for Ludwigs angina. It’s a combination of clinical examination, symptoms and radiographic examination.** **The patient reported hyperthermia (100° F), pulse rate of 99 beats per minute, respiratory rate of 25 breaths per minute, and oxygen saturation fluctuating between 88 to 90 SP02. On clinical examination, he reported a restricted mouth opening of only 19 mm (about 0.75 in), a tongue deviation [and evident dysphagia and dyspnea. Extra orally, the swelling was indurated, non-fluctuant, with bilateral involvement of the submandibular and sublingual spaces. His dentate status revealed root stumps in relation to 36 and 46 regions, which were tender on percussion. The obliteration of the mucogingival junction was also noted. Radiographic examination with orthopantomogram (OPG) revealed periapical radiolucency concerning 36,37 and 38 with locules in the submandibular region (more on the right side than the left side), indicating air or pus**

 7.No details have been provided regarding the utilization of laboratory tests and radiographic imaging in the assessment.

**Answer:** **Radiographic examination with orthopantomogram (OPG) revealed periapical radiolucency concerning 36,37 and 38 with locules in the submandibular region (more on the right side than the left side), indicating air or pus. The pus was collected with a sterile swab and sent for culture and antibiotic sensitivity test. The culture sensitivity report revealed the sensitivity to prescribed empirical antibiotics; therefore, the same medicines were continued for a week.**

 8.What are the respective roles of the first and second authors in the execution of this research?

**Answer: The first author did the surgical procedure while the second author provided assistance.**

 9.Was consent obtained from the patient prior to the capture of clinical photographs?

**Answer: yes. We obtained consent for treatment and for capturing clinical photographs.**

# Response

**Published:** 2024-05-21

**Authors:** Siddharthan Selvaraj

**Affiliations:** Faculty of Dentistry, University of Malaya, Kuala Lumpur, Federal Territory of Kuala Lumpur, Malaysia

1.The authors have failed to reference any established protocols for the management of Ludwig's angina.

**Answer:  Although there are no current guidelines for managing acute Ludwig's angina, the mainstay of the treatment includes airway maintenance and following the principles of infection control which we have already discussed.**

 2.What course of action should be pursued if the administration of steroids and epinephrine are contraindicated for a case of Ludwig's angina?

**Answer: Maintaining patent airway is the most important consideration. Removing the source of infection, antibiotics and Incision and drainage would be course of action .**

 3.The authors have neglected to include citations of recent publications. Is Ludwig's angina the most perilous type of facial and submandibular space infection?

**Answer: yes, it is highly perilous but in this antibiotic era it has become a rare infection which makes recent publications little difficult.**

 4.Justify the designation of 'dangerous space' in this context.

**Answer:** **It can either lead to airway obstruction or, at a later stage, cause dissemination of the infection, which then spreads through several other adjacent spatial planes.**

 5.The primary cause for the development of this condition in a 40-year-old male remains unspecified.

**Answer: yes the patient wasn’t immuno compromised or medically compromised at the time of diagnosis and it still happened**

 6.Which specific diagnostic procedures were employed in the examination of this particular case?

**Answer: There is no specific diagnostic test which gives the diagnosis for Ludwigs angina. It’s a combination of clinical examination, symptoms and radiographic examination.** T **he patient reported hyperthermia (100° F), pulse rate of 99 beats per minute, respiratory rate of 25 breaths per minute, and oxygen saturation fluctuating between 88 to 90 SP02. On clinical examination, he reported a restricted mouth opening of only 19 mm (about 0.75 in), a tongue deviation [and evident dysphagia and dyspnea. Extra orally, the swelling was indurated, non-fluctuant, with bilateral involvement of the submandibular and sublingual spaces. His dentate status revealed root stumps in relation to 36 and 46 regions, which were tender on percussion. The obliteration of the mucogingival junction was also noted. Radiographic examination with orthopantomogram (OPG) revealed periapical radiolucency concerning 36,37 and 38 with locules in the submandibular region (more on the right side than the left side), indicating air or pus**

 7.No details have been provided regarding the utilization of laboratory tests and radiographic imaging in the assessment.

**Answer:** **Radiographic examination with orthopantomogram (OPG) revealed periapical radiolucency concerning 36,37 and 38 with locules in the submandibular region (more on the right side than the left side), indicating air or pus. The pus was collected with a sterile swab and sent for culture and antibiotic sensitivity test. The culture sensitivity report revealed the sensitivity to prescribed empirical antibiotics; therefore, the same medicines were continued for a week.**

 8.What are the respective roles of the first and second authors in the execution of this research?

**Answer: The first author did the surgical procedure while the second author provided assistance.**

 9.Was consent obtained from the patient prior to the capture of clinical photographs?

**Answer: yes. We obtained consent for treatment and for capturing clinical photographs.**

# Reviewer report: Reviewer response for version 1

**DOI:** 10.5256/f1000research.139725.r158065

**Published:** 2022-12-22

**Authors:** Abedelmalek Tabnjh

**Affiliations:** 1 Department of Applied Dental Sciences, Jordan University of Science and Technology, Ar-Ramtha, Jordan

This paper is a well prepared case report. In the introduction the authors were covered the Ludwig's angina's definition, history, etiology, risk factors and the objective of the report. Then, they described patient's details, symptoms, diagnostic procedure, and treatment. The discussion section covered all aspects of the topic very well with good details.

 Just one point for improving:

*It will be better if the authors added if the patient had any risk factors or not. Yes they mention that he didn't have any medical history, but what about other risk factors such as alcoholism and malnutrition?*

 Over all this is a good case report.

Are enough details provided of any physical examination and diagnostic tests, treatment given and outcomes?

Yes

Is the case presented with sufficient detail to be useful for other practitioners?

Yes

Is sufficient discussion included of the importance of the findings and their relevance to future understanding of disease processes, diagnosis or treatment?

Yes

Is the background of the case’s history and progression described in sufficient detail?

Yes

Reviewer Expertise:

Oral Epidemiology, Biostatistics, Oral Hygiene, and Dental Materials.

I confirm that I have read this submission and believe that I have an appropriate level of expertise to confirm that it is of an acceptable scientific standard.
